# Identifying accessible prognostic factors for breast cancer relapse: a case-study on 405 histologically confirmed node-negative patients

**DOI:** 10.1186/s12957-017-1272-7

**Published:** 2017-11-23

**Authors:** Ines Zemni, Montassar Ghalleb, Ichraf Jbir, Maher Slimane, Jamel Ben Hassouna, Tarek Ben Dhieb, Hatem Bouzaiene, Khaled Rahal

**Affiliations:** Surgical oncology department, Institute Salah Azaiez of Oncology, Boulevard 9 avril 1938 Beb Saadoun, 1006 Tunis, Tunisia

**Keywords:** Breast cancer, Early stage, Node negative, Prognoses

## Abstract

**Background:**

Histologically, node-negative breast cancer generally have a good prognosis. However, 10 to 30% of the cases present local relapses or metastasis. This group of people has high chances of remission if detected early. The aim of this study is to identify financial affordability for developing countries to adjust treatment.

**Methods:**

We selected 405 patients with histologically confirmed node-negative breast cancer in our institution between January 2001 and December 2003. Patients with metastasis were excluded. The statistical analysis was conducted using SPSS ver. 18 (SPSS, Inc., Chicago, Illinois).

**Results:**

The medial age was 51 years old. The medial tumor size was 35.4 mm. Clinically, 67.2% of the patients were staged cT2 and 63.2%, cN1i. Breast conservation was achieved in 41% of cases. In the histologic examination, the medial size was 30 mm. Grade III tumors were found in 50.1% of patients and positive hormonal receptors in 53.4%. The mean number of lymph nodes was 14. Eight patients had neoadjuvant chemotherapy. Adjuvant locoregional radiation and adjuvant chemotherapy were prescribed respectively in 70.6 and 64.4% of cases. 59.7% had adjuvant hormonal therapy. The follow-up showed 17.7% cases of relapse either locally or in a metastatic way in a mean time of 57.4 months. The disease-free survival at 5 years was 82.1%, and the overall survival for the same period was 91.5%.

The histologic tumor size and the grade and number of lymph node dissected were shown to be influencing the disease-free survival. Radiation therapy and hormone therapy showed improved disease-free survival and overall survival.

**Conclusion:**

Our study found interesting results that may help personalize the treatment especially for patient living in underdeveloped countries, but further studies are needed to evaluate those and more accessible prognostic factors for a more accessible healthcare.

## Background

In developed country, the histologically node-negative breast cancer (HNNBC) represents two thirds of invasive breast cancer [1]. In Tunisia, it is less frequently seen and it accounts to 41.7% [2]. With the evolution of screening methods, more and more patients are diagnosed in early stage and without lymph node involvement. In developing countries, however, these are not always available.

HNNBC patients usually have a good prognosis. Of these women, 85% are expected to be alive and free from distant metastasis at 10 years [3]. Unfortunately 10 to 30% tend to either relapse locally or develop metastasis during the 10 years post diagnosis [1]. HNNBC are a heterogeneous group of tumors with different relapse abilities, doubling times, and different infiltrative capacities.

This led researchers to actively look for prognostic factors which can help personalize the treatment to every patient based on his overall risk to relapse.

In the era of multigene assays and biomarkers, risk evaluation is becoming more efficient. However, this new technologies remain expensive and often times are not available in some developing countries.

Throughout this study on 405 patients, we aimed to find statistically significant prognostic factors influencing the disease-free survival (DFS) and the overall survival for the HNNBC patients.

## Methods

We designed a retrospective cohort study involving 405 women seen in the Salah Aziez institute of oncology (Tunis) between January 2001 and December 2003 with HNNBC and no metastasis. Patients having ductal carcinoma in situ and initially metastatic breast cancer were excluded.

Cases were staged using TNM classification. Initial treatments were specifically made depending on the initial stage of the tumor. Treatment planning was made in multidisciplinary meetings.

The different possibilities of initial planning were as follows:

(A). Upfront surgery: radical mastectomy or breast-conserving surgery.
(B). Neoadjuvant chemotherapy followed by surgery (radical mastectomy or breast-conserving surgery).

No fine-needle biopsy or sentinel lymph node dissection was made due to the unavailability of these techniques at the time of the study.

The histological examination focused on tumor histotype, grade, and size; the number of tumor loci; the lymphovascular invasion; and the number of dissected lymph nodes, and immunochemistry data at the IHC included only hormone receptors due to the unavailability of HER2neu and KI67% in Tunisia at that time. Adjuvant treatment was also decided in a multidisciplinary meeting, with patient having either one or a combination of radiation therapy, chemotherapy, or hormone therapy.

Potential prognostic factors considered during the follow-up were as follows: age, menopausal status, clinical and histological size, tumor grade, clinically node positive, lymphovascular invasion, treatment modalities, immunochemistry data, and the number of dissected lymph nodes.

All statistical analysis was performed with SPSS ver. 18 (SPSS, Inc., Chicago, Illinois). We first ran a descriptive statistics of all potential risk factors. We then performed a bivariate analysis with a Pearson’s chi-square or a Fisher’s exact tests. Significant variables were then included in a multivariable Cox’s regression model. Significance was set at *p* value inferior or equal to .05, and both univariate and bivariate tests were two-tailed.

## Results

From a total of 1052 women seen for breast cancer between January 2001 and December 2003 in the Salah Aziez institute of oncology in Tunis, 405 patient were confirmed HNNBC. Those 405 patients were included in our study.

**Fig. 1:**
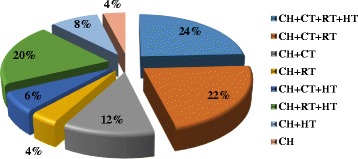
Distribution of patients according to treatment modalities

The medial age was 51 years old, and the medial tumor size was 35.4 mm. Clinically, 67.2% of the patients were staged cT2 and 63.2%, cN1. The different treatment modalities have been decided in a multidisciplinary meeting (Fig. 1).

Breast conservation was achieved in 41% of cases (166 patients).

**Fig. 2:**
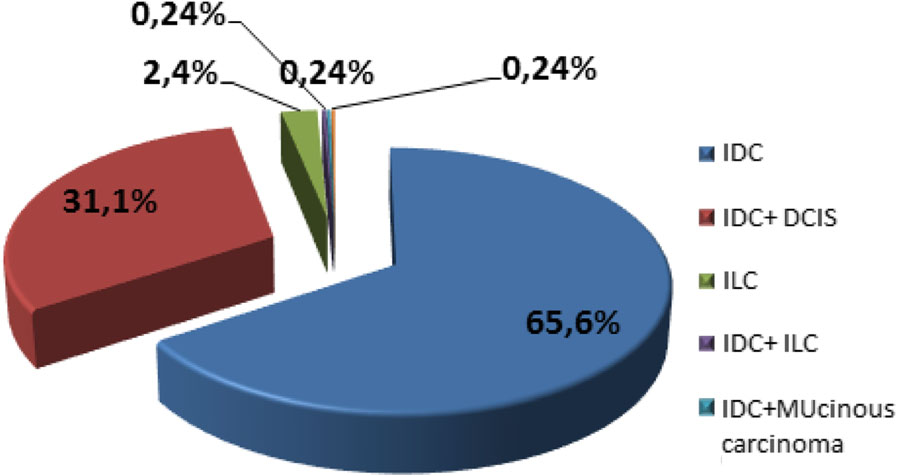
Distribution of patients according to the histologic type

The histological medial tumor size was 29.58 mm (6–90 mm). The most frequent histological type was invasive ductal carcinoma (IDC) (Fig. 2).

Most of tumors were SBR grade 3 (50.1%) and 2 (31.5%). Lymphovascular invasion was found in 17 patients (4.2%).

The medial number of lymph node dissect was 14 (4–44) and were all disease free.

The hormone receptors were tested in 290 patients (71.6%), and they were positive in 155 patients (53.4%) (Table 1).

**Table 1:** Patients distribution according to hormone receptors

		RP		Total
		Negative	Positive	
RO	Negative	135	19	154
	Positive	33	103	136
Total		168	122	290

The HER2neu receptors were tested in 38 patients (9.3%). An overexpression was found in 20 patients. It was not always tested due to the absence of anti-HER2neu treatment at the time of the study.

The medial time of follow-up was 57.4 months. Three hundred thirty-three patients were clinically and radiologically disease free.

A locoregional or metastatic relapse was seen in 79 patients (17.7%). Ninteen patient relapsed locally, 50 suffered a metastatic relapse and 10 suffred both metastatic and local relapse.

The overall DFS was 82.1% at 5 years of follow up. The different prognostic factors studied were as follows: age, menopausal status, clinical tumor size, T stage, clinical N stage, histologic tumor size, SBR grade, lymphovascular invasion, number of dissected lymph nodes, hormone receptors, HER2neu status, and treatment modalities (Table 2).

**Table 2:** Statistical significance of the studied prognostic factors on DFS

Hormone therapy	Chemotherapy	Radiation therapy	HER2neu status	Hormone receptors	Number of dissected lymph nodes	Lymphovascular invasion	SBR grade	Histologic tumor size	Clinical N stage	Clinical tumor size	Menopausal status	Age	
Yes	No	Yes	No	No	Yes	No	Yes	No	No	No	No	No	Significance on DFS
*p* = 0.011	*p* = 0.295	*p* = 0.036	*P* = 0.729	*p* = 0.959	*P* = 0.038	*P* = 0.161	*P* = 0.036	*p* = 0.053	*p* = 0.3	*p* = 0.553	*p* = 0.978	*p* = 0.982	*P*

The OS was 91.5% of patients at 5 years. 

Only patients receiving radiation therapy (*p* = 0.001) and hormone therapy (*p* = 0.001) showed improved OS.

## Discussion

HNNBC accounts for approximately two thirds of breast cancer in developed countries [1].

In Tunisia, according to the national cancer registry and Maalej et al.’s retrospective study, it accounts for 40% of the breast cancer [2, 4]. Our results with 38.5% of HNNBC between 2001 and 2003 confirm and come along with these findings.

The HNNBC is known for having a good prognosis. The OS for node-negative patients in the meta-analysis by the Early Breast Cancer Trialist’s Collaborative Group at 5 years was 81 to 89% of patients [5]. Here, despite the absence of taxane, aromatase inhibitors (AI), and trastuzumab, the OS was similar to the literature [6–8] and also to results found in studies using taxanes, AI, and trastuzumab [9–11].

The DFS at 5 years in the literature varies from 75.8 to 93.4% [6, 8, 10, 11] which is in accordance with our study.

However, in 10 to 30% of cases, it tends to relapse [1]. This led researchers to look for predictive factors of relapse for node-negative breast cancer in order to adjust the adjuvant treatment and long-term follow-up to the individual.

In the last years, “the risk predictive” armament were developed due to the emergence of multigene assays (MA) and molecular biomarkers (MC).

This new technology has proven its efficacy but it remains expensive and not available in all countries.

### Age

In the Saint Gallen consensus, since 1998, women under the age of 35 years old were known to have worse prognoses than the other age categories [12].

Kuru et al. [13], in their 384 patients’ study, found that women under 35 years old had a worse prognoses than those aged between 35 and 49 years old. The difference was significant for both OS (*p* = .007) and DFS (*p* = 0.01).

Wang et al. [14] also showed, in their 62 patients’ study, that women aged under 35 years old had a worse DFS.

Other authors did not found age-significant prognostic factors influencing OS and DFS [15, 16]. In our study, age was not found to be significantly influencing the OS and DFS.

### Menopausal status

In most studies, it did not appear as a significant prognostic factor [8, 14, 16, 17].

Jagsi et al. and their 877 cases study found a worse DFS for premenopausal women [18].

In our 405 cases study, menopausal status did not influence OS or DFS.

### Clinical tumor size

Most of the studies were interested in the histologic tumor size and not in the clinical.

Kato et al. and Chevallier et al. found that the clinical tumor size is a strong prognostic factor influencing OS [19, 20].

In our work, with a *p* = 0.557, it was nearly significant.

### Histologic tumor size

A histologic size superior to 2 cm was found to be a prognostic factor for the high-risk group defined by the Saint Gallen group [12]. In the 2009 update, for a size superior to 5 cm, chemotherapy was indicated [21].

Some study compared DFS and/or OS between patients with tumors inferior to 2 cm and others with more than 2 cm, and they showed significant results [8, 13, 14, 16, 22].

Trudeau et al. [15] found while comparing three groups of patients (< 2 cm, 2 to 5 cm, and > 5 cm) that size influenced both DFS and OS (*p* = 0.001).

In this study, histologic tumor size was found to be influencing DFS but not OS.

### Tumor grade

In Wang et al. study [14], grade was not a significant prognostic factor. However, in many other studies, it was found to be a strong prognostic factor influencing both OS and/or DFS [13, 15, 22, 23].

Our work showed a significance on DFS but not for OS.

### Number of dissected lymph nodes

With the development of sentinel lymph node biopsy (SLB) and screening techniques, more and more women can be diagnosed at an early stage. It may seem odd to discuss this prognostic factor. However, in some developing countries where these methods are often unavailable or out of reach, there are still some women who need axillary lymph node dissection to fully stage the disease.

Blancas et al. [8], 1606 case series, found that women with more than six dissected lymph nodes (DL) had a better DFS with a *p* = 0.014, and they concluded that an insufficient number of DL should be considered as a pejorative prognostic factor and taken into account while discussing the adjuvant treatment.

Tai et al. [23] compared two groups of patients. One treated with both conservative surgery and radiation therapy. The second treated only with conservative surgery. They found that in the second group (the group with surgery alone), a number of DL superior to 10 was associated with a better OS. While in the first group (surgery and radiation therapy), no impairment of OS was noted. The authors concluded that radiation therapy can be a good option in patients with low number of DL.

Some authors [24, 25] did not find any impact of DL on DFS and concluded that there is no high-risk group of relapse based on number of DL.

Mersin et al. [26] showed that an extensive dissection of the axilla (DL superior to 18) can impair both OS and DFS.

Our results showed an impact on the DFS but not in the OS.

### Lymphovascular invasion

The lymphovascular invasion (LI) was proved in many studies to be an independent prognostic factor influencing both OS and DFS [13, 16, 17, 27, 28].

For Lee et al [27], LI was founded to be a strong prognostic factor.

In the 2007 Saint Gallen Guidelines, the lymph vascular invasion was added as a prognostic factor to take into account while discussing systemic therapy [29].

In our study, the percentage of patients with LI was low (4.2%); this can explain why it did not have an impact on both DFS and OS.

### Hormone receptors

Bull et al. [7] found that hormone receptors (RH) influenced both DFS and OS in a univariate analysis and that negative RH was a bad prognostic factor.

Some other authors found the same conclusion [8, 17, 22].

In Trudeau et al.’s [15] uni- and multivariate analysis, RH did not impair both OS and DFS (estrogen receptors, *p* = 0.7; progesterone receptors, *p* = 0.61). Similar results were found in other studies [14, 16, 30].

Our results join those of the latter group of authors as RH did not influence OS and DFS.

### HER2 status

Overexpression of HER2 is a negative prognostic and predictive risk factor for survival; however, with the advent of trastuzumab, patients’ prognosis is improving in all treatment settings [31].

Chia et al. [32], in their study including 2026 HNBCC with 206 patients overexpressing HER2, showed that the 10-year OS for HER2-negative patients was better (74.4 vs 65%, *p* = 0.06).

Tovey et al.’s [33] study with 362 patients found similar results with a worse 5-year cancer-specific survival in patients with overexpression of HER2 (68 versus 96%; *p* < 0.001).

Other studies did not found the overexpression of HER2 as a significant prognostic factor especially for small-sized tumors [30].

In our work, due to the absence of trastuzumab, looking for the overexpression of HER2 was not routinely done and this can explain why it did not have an impact on both DFS and OS.

### Adjuvant treatment

The effect of adjuvant treatment on OS and DFS was added to the “Results” section in order to show their positive impact for HNNBC in carefully selected patients, to emphasize the purpose of this work which is to find more prognostic factors that will help select patients that will benefit from an adjuvant treatment.

## Conclusion

The HNNBC is becoming more and more frequent with the development of the screening techniques.

It usually carries good prognoses but, in some cases, tends to relapse. This led the science in a quest to assess the risk of this population in order to offer the best treatment to the patients. Many advances in genetics paved the way to risk assessment; however, it remains expensive and not widely available, which led us through this work to try and find more accessible prognostic factors to assess the risk of relapse.

The histologic tumor size and the grade and number of lymph node dissected were shown to be influencing the disease-free survival.

Further studies are needed to evaluate those and more accessible prognostic factors for a more accessible healthcare.
